# Association of Angiotensin-Converting Enzyme (ACE) Gene Polymorphism with Inflammation and Cellular Cytotoxicity in Vitiligo Patients

**DOI:** 10.1371/journal.pone.0132915

**Published:** 2015-07-15

**Authors:** Laila Rashed, Rania Abdel Hay, Rania Mahmoud, Nermeen Hasan, Amr Zahra, Salwa Fayez

**Affiliations:** 1 Biochemistry Department, Faculty of Medicine, Cairo University, Cairo, Egypt; 2 Dermatology Department, Faculty of Medicine, Cairo University, Cairo, Egypt; 3 Biochemistry Department, Fayoum University, Fayoum, Egypt; National Research Council of Italy (CNR), ITALY

## Abstract

**Background:**

Vitiligo is a disorder with profound heterogeneity in its aetio-pathophysiology. Angiotensin converting enzyme (ACE) plays an important role in the physiology of the vasculature, blood pressure and inflammation. An insertion/deletion (I/D) polymorphism of the ACE gene was reported be associated with the development of vitiligo.

**Objective:**

Our aim was to evaluate the ACE I/D polymorphism in vitiligo patients and controls. Our second aim was to find a possible association between ACE gene polymorphism and inflammatory mediators (as interleukin (IL)-6) and/or cellular cytotoxicity induced by serum nitrite (as a breakdown product of the cytotoxic nitric oxide) in vitiligo patients.

**Methods:**

This case-control study included 74 vitiligo patients and 75 apparently healthy controls. The distribution of ACE gene I/D genotype was investigated using PCR. Serum ACE, IL-6 and nitrite were measured by colorimetric method, ELISA and Griess assay respectively.

**Results:**

The ACE allele frequency was significantly different between vitiligo patients and healthy controls (P = 0.026). However there was no significant difference between the ACE genotyping frequency in both groups (P = 0.115). There were statistically significant higher VIDA score (P = 0.007), and serum IL-6 (P < 0.001) in patients with the DD genotype when compared to other genotypes. Serum nitrite in patients with the DD genotype was significantly higher (P = 0.007) when compared to patients with II genotype. Serum levels of ACE, IL-6 and nitrite in vitiligo patients were statistically significantly higher than those in controls.

**Conclusion:**

As a conclusion, ACE gene polymorphism might grant susceptibility to develop vitiligo. Serum IL-6 and nitrite levels might have an important role in the pathogenesis of vitiligo. Targeting these two factors might have an implication in the treatment of some resistant cases.

## Introduction

The associations of vitiligo with other autoimmune diseases together with the detection of anti-melanocyte and other antibodies in vitiligo patients led to the proposal of the theory of melanocyte autoimmune destruction in vitiligo [1]. Studying the polymorphisms of certain genes incorporated in the immune system detected a significant role in vitiligo susceptibility [2,3].

Angiotensin converting enzyme (ACE) is an important regulator of the renin-angiotensin system (RAS) and kallikrein-kininogen systems by creating angiotensin II (Ang II) and inactivating bradykinin. ACE has a significant role in the physiology of the blood vessels and inflammatory process and it has been widely studied to detect its association with various autoimmune diseases [4].

An insertion/deletion (I/D) polymorphism in intron 16 of the ACE gene accounts for most of the variability of serum ACE activity and is associated with the development of vitiligo. The D allele appears to grant susceptibility to vitiligo [4].

Among the different effects of Ang II, it increases both the generation of reactive oxygen species (ROS) and the synthesis of cytokines such as interleukin-6 (IL-6) and IL-8, thus exerting proinflammatory effects [5]. ACE inactivates bradykinin which promotes vasodilation by enhanced formation of nitric oxide (NO), increases vascular permeability, and stimulates the synthesis of proinflammatory cytokines such as IL-6 and IL-8 [6]. Both IL-6, as a proinflammatory cytokine, [7–9] and NO, as a cytotoxic agent, [10–12] have been suggested to play a role in the pathogenesis of vitiligo. To the best of our knowledge no previous studies have examined the possible association between serum IL-6 (as an inflammatory mediator) and serum nitrite (breakdown product of NO, as a cytotoxic agent) with the different ACE gene polymorphism in vitiligo.

The aim of the present study was to investigate a hypothesized association between the ACE gene polymorphism and the presence of vitiligo. We aimed also to find a possible interplay between ACE gene polymorphism with the inflammatory mediator IL-6 and/or cellular cytotoxicity induced by serum nitrite in vitiligo patients.

## Materials and Methods

This case control study included 74 vitiligo patients consecutively recruited from the dermatology outpatient clinic, Kasr Al-Aini Hospital, Cairo University, Egypt. Patients suffering from any other skin or autoimmune disorders had been excluded. As a control group, 75 apparently healthy age and sex matched volunteers without any clinical evidence or family history of vitiligo or any other skin and autoimmune disorders were included in the study. The study was approved by the research ethical committee (REC) of Dermatology department, Faculty of Medicine, Cairo University. Written informed consent that has been approved by the REC has been signed by all adult subjects and by guardians of enrolled children in the study, and then kept with us for documentation.

Both groups were subjected to detailed history taking and clinical assessment was done to determine the type, and extent of vitiligo, and to exclude vitiligo in the control group.

### Detection of ACE polymorphism

**Fig 1:**
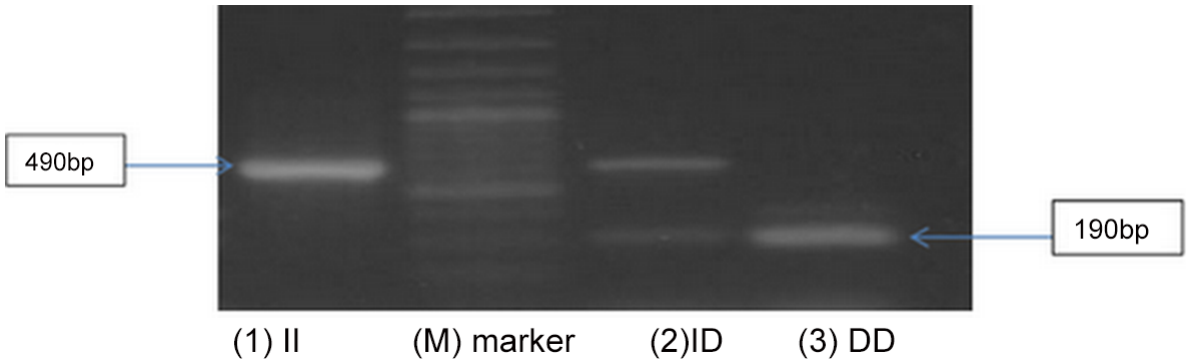
The complete gel image with all different size of bands of ladder; Lane M: DNA ladder with 100 bp, Lane1: ACE genotype (II) with PCR products 490 bp, Lane 2: ACE genotype (ID) with PCR products 490&190 bp, Lane 3: ACE genotype (DD) with PCR products 190 bp.

DNA samples were extracted from the whole blood of the cases and controls using DNA extraction kit (Qiagen Inc, Germany) according to the manufacturer’s protocol. The amount of the DNA was quantified and the quality of the DNA was determined.Amplification was carried out in a DNA thermocycler [Biometra cycler]. PCR was performed using 20 pmoles of each primer (flanking primer pair): Sense oligo 5′ - CTG GAG ACC ACT CCC ATCCTT TCT 3′ and anti-sense oligo: 5′ -GAT GTG GCC ATC ACA TTC GTC AGA T-3′ in a final volume of 25μl, containing (0.5 μ g genomic DNA, 2mM MgCL2, 50mM KCL, 10mM Tris-HCL (pH = 8.3), 0.2 mM of each dNTP, and 0.5 unit of Taq polymerase. PCR was done with an initial denaturing time at 94°C for l min. Then the DNA was amplified for 30 cycles with denaturation at 94°C for 30 s, annealing at 58°C for 30 s, and extension at 72°C for l min. This was followed by final extension at 72°C for 10 min. PCR products were directly visualized using ethidium bromide staining after electrophoresis in a 2% agarose gel [13]. The amplification product is a 190 bp fragment in the presence of the deletion (D) allele and a 490 bp fragment in the presence of the insertion (I) allele. Therefore, there were three genotypes after electrophoresis: A 490 bp band (genotype II), 190 bp band (genotype DD), or both 490 and 190bp band (genotype ID) (Fig 1).

### Determination of nitrite

Measurement of the NO end product nitrite production was used to assess relative values of the NO. Nitrites were determined by the standard Griess assay [11].

### Detection of IL-6

Interleukin-6 level was determined using a commercially available ELISA kit (Quantikine, human IL-6R & D Systems, Minneapolis, USA) in accordance with the manufacturer’s instructions [14].

### Measurement of ACE Level

ACE level was measured by a colorimetric method (colorimetric assay kit, Fujizoki Assay, Tokyo, Japan) using p-hydroxyhippuryl- L-histidyl-L-leucine as the substrate [15].

### Statistical analysis

Data was coded and entered using the statistical package SPSS (Statistical Package for the Social Science; SPSS Inc., Chicago, IL, USA) version 15. Data was summarized using mean, standard deviation and range for quantitative variables. Comparison between two groups was done using independent sample t-test or ANOVA (analysis of variants) for comparing more than two groups. The frequencies of the I/D alleles and genotypes were compared between vitiligo patient and control groups using chi-square test. Correlations were done to show the relation between quantitative variables using Pearson’s coefficient. P-values < 0.05 were considered to be statistically significant.

## Results

### Demographic data

(See S1 Table.)

This case control study included 74 vitiligo patients (27 males and 47 females); their age ranged from 5 to 68 years with mean age 31.5 years, their disease duration ranged from 0.08 to 12 years with mean 4.36 years. The clinical presentation of the vitiligo group included 3% (n = 2) presenting with focal type, 5% (n = 4) with segmental type, 8% (n = 6) with acral type, 70% (n = 52) with vulgaris type, 11% (n = 8) with mixed type and 3% (n = 2) with universal vitiligo. Previous treatment was received by 80% (n = 59/74) of patients, and response to treatment in those cases revealed no response in 12/74 (16.2%), poor response in 29/74 (39.2%) of patients, mild response in 2/74 (2.7%) of patients and good response in 16/74 (21.6%) of patients. The 75 age and sex matched healthy controls were 32 males and 43 females; their age ranged from 13 to 70 years with mean age 34 years, they had no clinical evidence or family history of vitiligo or of any other autoimmune disorder (Table 1).

**Table 1:** Summary of the demographic and biochemical data of the vitiligo patients and controls.

Data	Patients (n = 74)	Controls (n = 75)	P-value
**Age/years (Mean±SD)**	31.54±15.44	34±13.80	0.302
**Male/Female (N,%)**	27 (36.5%)/47 (63.5%)	32 (43%)/43 (57%)	0.441
**Extent of disease (% of body, Mean±SD)**	22±19.40		
**Duration/years (Mean±SD)**	9.61±19.24		
**Positive family history of vitiligo (N,%)**	12/74 (18%)		
**Positive history of associated stress (N,%)**	40/74 (54%)		
**VIDA score (Range, Mean±SD)**	0–4 (2.40±1.41)		
**Serum ACE (IU/l) (Range, Mean±SD)**	10.5–32.4 (24.39±5.25)	9.60–20.1 (12.59±3.13)	<0.001*
**Serum IL-6 (pg/ml) (Mean±SD)**	(81.85±17.33)	29.12±4.15	<0.001*
**Serum nitrite (nmol/l) (Mean±SD)**	43.26±12.87	11.6±2.45	<0.001*

SD, standard deviation; VIDA,vitiligo disease activity score; ACE, angiotensin converting enzyme; IL-6, interleukin-6;

* *p* value <0.05 is statistically significant.

### Biochemical data and genotype distribution

It was observed that serum ACE, serum IL-6 and serum nitrite were statistically significant higher in patients than in controls (P < 0.001). The genotypic distribution of ACE gene polymorphism and allelic frequency for I and D alleles in patients and controls have been given in Table 2. It was observed that patients showed a comparatively much higher percentage of the ID genotype 37.8%, compared to 31.1% for each of DD and II genotypes. Among the controls, the rate of occurrence of the II genotype showed a frequency of 46.7%, while the ID and DD genotypes were found to be 33.3% and 20.0% respectively. The studied populations follow the Hardy-Weinberg Equilibrium. The ACE gene genotype distribution showed no significant difference between patients and controls (P = 0.115) while the allele frequency was significantly different between patients and controls (P = 0.026). The results indicated that the D allele was significantly over-represented in patients compared with controls (50% Vs 36.7%) (Table 1).

**Table 2:** Comparison of ACE genotyping and allele frequency between patients and controls.

Genotype	Patients (N, %)	Controls (N, %)	Odds ratio with 95% confidence interval	P-value
**I/I**	23/74 (31.1%)	35/75 (46.7%)	1.940 (0.993–3.789)	0.115
**I/D**	28/74 (37.8%)	25/75 (33.3%)	0.821 (0.420–1.608)	0.115
**D/D**	23/74 (31.1%)	15/75 (20%)	0.554 (0.262–1.174)	0.115
**Allele frequency**				
**I**	74/148 (50%)	95/150 (63.3%)	1.727 (1.087–2.744)	0.026*
**D**	74/148 (50%)	55/150 (36.7%)	0.579 (0.364–0.920)	0.026*

N, number;

*p value <0.05 is statistically significant.

### Studying the different variables in relation to the demographic data

Studying the different variables as regards the sex difference and the presence of positive family history in the patients group showed a non^-^significant difference.

The different studied quantitative variables were compared regarding the association of any stressful event with development of vitiligo in our patients and revealed a statistically significant difference as regards the extent of disease (P = 0.041) with a higher extent being detected in patients with associated stress. Other variables such as VIDA score, serum IL-6 and serum nitrite showed a non-significant difference (Table 3). The age of the patients, the VIDA score, the duration of the disease, the serum IL-6 and the serum nitrite have been compared in different clinical types of vitiligo and showed no significant difference (Table 4). We didn’t find any statistical significant difference in the serum IL-6 and nitrite when comparing treated versus untreated patients (P = 0.472 and 0.403 respectively).

**Table 3:** Comparison between the different variables in vitiligo patients regarding the association of stress.

Variables (Mean±SD)	Negative association with stress (n = 34)	Positive association with stress (n = 40)	P-value
**Extent of disease (%)**	17.02±11.93	26.22±23.33	0.041*
**VIDA score**	2.50±1.39	2.32±1.44	0.599
**Serum IL-6 (pg/ml)**	80.72±16.96	82.82±17.79	0.606
**Serum nitrite (nmol/l)**	43.31±13.69	43.23±12.29	0.979

SD, standard deviation; n, number; VIDA,vitiligo disease activity score; ACE, angiotensin converting enzyme; IL-6, interleukin-6;

* *p* value <0.05 is statistically significant.

**Table 4:** Comparison between the studied variables in different vitiligo clinical types in our patients.

Variables (Mean±SD)	Focal type (n = 2)	Segmental type (n = 4)	Acral type (n = 6)	Vulgaris type (n = 52)	Mixed type (n = 8)	Universal type (n = 2)	P-value
**Age (years)**	36±1.41	18±3.56	29.66±21.81	31.65±15.57	35.25±14.71	41±2.83	0.484
**Extent of disease (%)**	12.5±10.61	6.25±2.50	8.16±5.98	21.8±16.67	26.25±9.16	92.5±3.53	<0.001*
**VIDA score**	3±1.41	1.5±1.73	2.83±1.17	2.57±1.38	1.75±1.39	0.5±0.71	0.118
**Disease duration (years)**	10.75±13.08	5.12±4.59	5.03±5.37	10.93±22.64	6.13±3.54	11±4.24	0.960
**Serum IL-6 (pg/ml)**	99.35±12.51	81.67±25.49	82.76±9.89	80.82±16.97	85.2±19.71	75.55±32.88	0.743
**Serum nitrite (nmol/l)**	54.64±0.78	45.54±17.96	45.75±11.67	41.99±12.76	46.09±13.01	41.80±21.92	0.738

SD, standard deviation; n, number; VIDA,vitiligo disease activity score; ACE, angiotensin converting enzyme; IL-6, interleukin-6;

* *p* value <0.05 is statistically significant.

**Fig 2:**
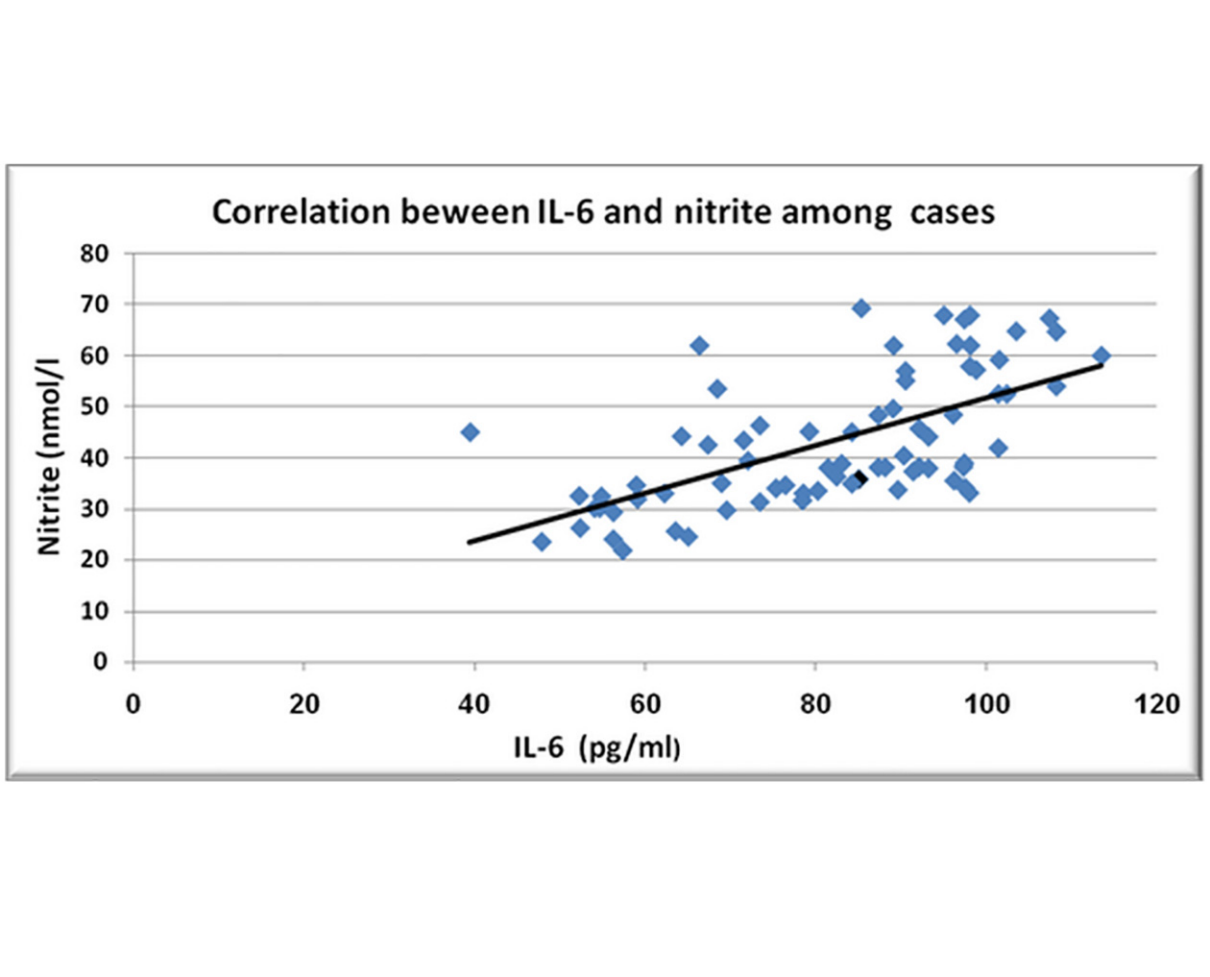
Scattered diagram showing the correlation between serum IL-6 and nitrite in vitiligo patients.

Our results revealed a significant negative correlation between VIDA score and the extent of disease in vitiligo patients (r = -0.249, P = 0.032), and a significant positive correlation between VIDA score and serum nitrite (r = 0.230, P = 0.048). A significant positive correlation between serum IL-6 and serum nitrite in both patients (r = 0.628, P < 0.001) and controls (r = 0.747, P < 0.001) has been also detected in this study (Fig 2).

### Studying the different variables in relation to the different ACE genotypes in patients

The analysis between the different ACE genotypes in vitiligo patients showed statistically significant higher VIDA score (P = 0.007), higher serum ACE (P < 0.001), higher serum IL-6 (P < 0.001) and higher serum nitrite (P = 0.007) in patients with the DD genotype, while the extent of disease did not differ statistically between the 3 groups (Table 5). Multiple comparisons between the different ACE genotypes regarding the different variables using post hoc testing revealed that VIDA score was significantly different only when comparing group I (II) to both groups II (ID) and III (DD), while serum ACE and IL-6 were significantly different when comparing each group to the other one, and serum nitrite was significantly different only when comparing group I to group III. The odds ratios (ORs) for the risk of vitiligo with 95% confidence intervals (Cis) have been be adjusted for age and gender when using multivariate logistic regression analyses.

**Table 5:** Comparison between the studied variables in different ACE genotypes in vitiligo patients.

Variables (mean±SD)	I/I (n = 23)	I/D (n = 28)	D/D (n = 23)	P-value
**Extent of disease (%)**	25±22.38	19.03±14.63	22.6±21.56	0.548
**VIDA score**	1.65±1.40	2.67±1.39	2.82±1.19	0.007*
**Serum ACE (IU/l)**	18.66±2	23.61±1.79	31.07±1.36	<0.001*
**Serum IL-6 (pg/ml)**	68.8±16.01	82.08±14.96	94.62±10.79	<0.001*
**Serum nitrite (nmol/l)**	36.94±12.80	44.08±11.58	48.60±12.18	0.007*

SD, standard deviation; n, number; VIDA,vitiligo disease activity score; ACE, angiotensin converting enzyme; IL-6, interleukin-6;

* *p* value <0.05 is statistically significant.

## Discussion

Serum IL-6 and serum nitrite play a role in the pathogenesis of vitiligo and the D allele of the ACE I/D gene polymorphism may confer susceptibility to vitiligo.

In the current study serum IL-6 was significantly higher in vitiligo patients when compared to healthy subjects. IL-6 is an inflammatory cytokine that can promote leukocyte-melanocyte interactions through the expression of ICAM-1 in the melanocytes that will further induce B-cell activation and autoantibody release, which ends up with melanocytes destruction. An increase in the production of IL-6 in vitiligo has a reported role in melanocytic cytotoxicity [16].

These results are consistent with others [8,9,16–18] who reported that the serum IL-6 was significantly elevated in vitiligo.

Our results revealed also that serum nitrite was significantly higher in vitiligo patients when compared to healthy subjects. NO has been previously found to have an autodestructive effect on normal human melanocytes [18] and it can reduce the attachment of melanocytes to the extracellular matrix leading to depigmentation [19]. Moreover, accumulation of 6 tetrahydrobiopterin (BH4), a co-factor in the synthesis of NO, in vitiligo [20] might be responsible for the high NOS activity and therefore high levels of NO in these cases. NO is a free radical and inherently reactive and mediate cellular toxicity by damaging critical metabolic enzymes [10]. So, NO plays a major role as a triggering agent in the pathophysiology of vitiligo [12].

These results are in agreement with others [17,21] who reported that there is an increase in the level of nitrite in vitiligo patients.

The results of the current study revealed the significant association of higher extent of disease in patients with preceding stressful event related to the onset of their disease. Certain events such as a death in the family, broken marriage, and other stressful conditions were found to precede and be associated with the onset of vitiligo [22]. Patients with vitiligo have a greater sensitivity to environmental stress and a lower threshold to generate catecholamine mediated responses. The precipitating factors might disturb the immunological balance of the body, leading to the paralysis of the affected patient’s melanocytes [23].

Our findings revealed a significant positive correlation between VIDA score and serum nitrite. This result suggests that serum nitrite could be used as a serum marker for the disease activity. Also the current study revealed a significant positive correlation between serum IL-6 and nitrite in vitiligo patients, suggesting a possible relation between these factors in the pathogenesis of vitiligo.

The results of the current study revealed that the ACE polymorphism of the I/I type was more frequent in controls than in vitiligo patients, while the I/D type and the D/D type were more frequent in vitiligo patients than in controls. However this difference was statistically non-significant and this can be explained by the small sample size of our study. These results are consistent with Tippisetty et al., [24] who reported that analysis of genotype frequencies revealed an over-representation of DD and ID among vitiligo patients, compared to that of the control group. This observation indicates increased susceptibility of the DD genotype to vitiligo while analysis of the II genotype frequency revealed a reduction in the frequency of this genotype among the patients compared to controls.

Our results revealed that the frequency of the I and D alleles was significantly different between the vitiligo patients and the controls, with the ACE I allele showing a frequency of 50% and of 63.3% in patients and controls, respectively. These results were consistent with others [4,24,25] who reported that the allele frequency is significantly different between vitiligo patients and healthy individuals. They reported that ACE DD genotype might be considered as a genetic risk factor to develop vitiligo in their studied population [25].

A protective role of the II homozygous of the ACE gene against the development of vitiligo has been suggested, as the II genotype is suggested to be less ROS-generating compared to other genotypes [24]. These results were in disagreement with others [3,26] who found no significant association between ACE gene polymorphism and vitiligo. Song et al., [26] suggested that the discrepancy between the results of different studies could be due to the small sample sizes of the studied population or due to the differences in ethnicity in the different studies [26].

The aetiopathogenesis of vitiligo involves complex interaction of environmental and genetic factors that contribute to melanocytes destruction. The studied genetic background of vitiligo categorized it as an autoimmune disease. Multiple studies have been performed to detect the involved genes [27]. The association of vitiligo with autoimmune disorders [28], the demonstration of autoantibodies to melanosomal proteins in vitiligo patients [29] and the proof that vitiligo autoantibodies can destroy pigment cells [30] support the autoimmune hypothesis in vitiligo.

In this study we observed a significant correlation of the serum ACE levels with the different ACE genotypes; patients with ACE DD genotype showed the highest mean ACE serum level, while patients with ACE II genotype had the lowest mean serum ACE level. Previous investigations have shown that elevated levels of ACE have been associated with autoimmune diseases [31] and an I/D polymorphism of the ACE gene accounts for most of the variability of serum ACE activity, with DD and II genotypes having the highest and the lowest activity, respectively. Thus ACE confers susceptibility to autoimmune disorders [32]. The ACE I/D polymorphism affects ACE activities; ACE DD genotype has been reported to increase serum ACE. ACE catalyzes the conversion of Ang I to Ang II [33]. Ang II increases both the generation of ROS and the synthesis of inflammatory cytokines such as IL-6 [5]. Oxidative stress induced by the generation of ROS can promote the development of vitiligo [12]. ACE has been also reported to degrade SP that promotes vasodilation, it also upregulates ICAM-1 expression in the endothelial cells, and promotes the expression of inflammatory cytokines such as IL-6 and IL-8 [33].

In this study, vitiligo patients showed a significant higher VIDA score, higher serum IL-6 and higher nitrite in patients with the DD genotype of ACE polymorphism. These results could suggest an association of vitiligo patients with the DD genotype of ACE polymorphism with more active disease. In support of these results, other studies [34,35] have demonstrated a significant increase in the frequency of the D allele of the ACE I/D polymorphism in vitiligo patients.

To the best of our knowledge this is the first study to detect a significant association between higher serum IL-6 and higher serum nitrite with the DD genotype of the ACE gene in vitiligo. However the small sample size of the studied population represents a limitation of the current study.

### Conclusion

Some cytokines like IL-6 that mediate many functions of the immune cells might play an important role in the pathogenesis of autoimmune diseases such as vitiligo. Free radicals like NO that occur during several physiological and pathological processes might also play a part in the pathogenesis of vitiligo. Targeting these two factors might have an implication in the treatment of some resistant cases. The D allele of the ACE I/D gene polymorphism in this study might confer susceptibility to develop vitiligo.

## Supporting Information

S1 Table All relevant data are within the Supporting Information file. (XLS)
